# A systematic review and network meta-analysis of the efficacy and safety of third-line and over third-line therapy after imatinib and TKI resistance in advanced gastrointestinal stromal tumor

**DOI:** 10.3389/fphar.2022.978885

**Published:** 2022-11-21

**Authors:** Xianhao Xiao, Weiye Yuan, Chong Wang, He Song

**Affiliations:** The First Hospital of China Medical University, Shenyang, Liaoning, China

**Keywords:** gastrointestinal stromal tumor, precise targeted therapy, refractory GIST, network meta-analysis, systematic review

## Abstract

Tyrosine kinase inhibitors (TKIs) have greatly improved the prognosis of unresectable and metastatic gastrointestinal stromal tumors (GISTs) in the last two decades. Imatinib and sunitinib are recommended as first-line and second-line therapies, respectively. However, there is a lack of precision therapy for refractory GISTs regarding therapy after imatinib and sunitinib. We comprehensively searched electronic databases, including PubMed, EMBASE, Web of Science, Cochrane Library, and ClinicalTrials, from inception to October 2022. Randomized controlled trials featuring comparisons with third-line or over third-line therapies against GISTs were eligible. The primary outcome was progression-free survival (PFS). All network calculations were performed using random effect models, and the ranking of regimens were numerically based on the surface under the cumulative ranking (SUCRA) statistics. A total of seven studies were eligible for inclusion in this network meta-analysis. After analysis, ripretinib was ranked at the top in progression-free survival (PFS), overall survival (OS), and disease control rate (DCR) (SUCRA statistics: 83.1%, 82.5%, and 86.5%, respectively), whereas nilotinib and pimitespib presented better tolerability (SUCRA statistics: 64.9% and 63.8%, respectively). We found that regorafenib seemed more reliable for clinical administration, and ripretinib showed good effectiveness for the over third-line therapy. Precise targeted therapy is a critical direction for the future treatment of GIST, and more high-quality studies of new agents are expected.

## Introduction

GISTs are the most common mesenchymal tumors associated with the gastrointestinal tract with a global incidence of 10–15 cases per million (Joensuu et al., 2002; Mucciarini et al., 2007; Serrano and George, 2014; Zhang et al., 2021). Most GISTs are genetically driven by KIT and PDGFRA mutations. Before the 21st century, GISTs were also known as gastrointestinal stromal sarcomas and demonstrated a high rate of primary resistance to chemotherapy and radiation that led to a poor overall prognosis (Hirota et al., 1998; Kim and Zalupski, 2011). However, imatinib (the first TKI approved by the US Food and Drug Administration (FDA) for therapy of GISTs) has revolutionized the therapeutic approach to GISTs and improved the prognosis of patients with advanced GISTs from a pre-imatinib 2-year overall survival (OS) rate of 25% to approximately 70–80% (Demetri et al., 2002; Verweij et al., 2004; Blanke et al., 2008; Le Cesne et al., 2010). Although imatinib resulted in an unprecedented efficacy and tolerance in the GISTs with a median progression-free survival (mPFS) of almost 2 years, most patients acquired secondary mutations and approximately 15% of the patients exhibited primary resistance to imatinib (Antonescu et al., 2005; Joensuu, 2006; Antonescu, 2008; Demetri, 2011). Secondary mutations that mainly occur in the ATP-bounding domain or activation loop blocked the binding of some TKIs and resulted in tumor progression (Liegl et al., 2008; Namlos et al., 2018).

Sunitinib, a multi-targeted TKI that inhibits KIT, PDGFRA, and vascular endothelial growth factor receptors (VEGFRs), is approved for the treatment of advanced GIST after the failure of imatinib (Demetri et al., 2006). In a phase III trial of advanced GISTs after the failure of imatinib, sunitinib displayed an mPFS of 22.9 weeks (Demetri et al., 2006; Demetri et al., 2012).

Even after receiving this second-line treatment, most patients continue to experience disease progression; these patients are considered refractory cases and require third-line or fourth-line treatment (regorafenib and ripretinib). The third-line (regorafenib) treatment demonstrated an mPFS of 4.8 months; this discouraging outcome probably indicates incomplete suppression of secondary resistant mutations.

Presently, there is no consensus on refractory therapies despite the National Comprehensive Cancer Network (NCCN) guidelines (version 1. 2021) that recommend sorafenib, dasatinib, nilotinib, pazopanib, and everolimus plus TKI as potential options for refractory cases.

Consequently, it is essential to simultaneously compare the efficacy and tolerance of multiple regimens for refractory cases. Recently, several novel TKIs, such as ripretinib, pazopanib, and pimitespib, have displayed promising superiority over the placebo (Mir et al., 2016; Blay et al., 2020; Kang et al., 2021). In this network review, we estimate and rank the efficacy and tolerance of all possible therapies for patients with advanced refractory GIST, which were previously treated with at least first- and second-line regimens, using a Bayesian network model.

## Methods

### Search strategy and selection criteria

In this systematic review, we researched patients with advanced gastrointestinal stromal tumors and that had progressed or were intolerant to at least two TKIs. The present meta-analysis was network designed. In this case, we did not set a specific intervention and comparator. We analyzed the outcomes of PFS, DCR, OS, and AE, and all included studies were RCTs.

We comprehensively searched electronic databases including PubMed, Web of Science, Embase, Cochrane Library, and ClinicalTrials. Our search terms included the following: gastrointestinal stromal tumor, gastrointestinal stromal neoplasm, gastrointestinal stromal sarcoma, imatinib, STI-571, imatinib methanesulfonate, glivec, randomized controlled trials, controlled clinical trials, randomized, etc. This detailed search strategy is listed in Supplementary File S1.

The search process covered possible trials published from inception to October 12, 2022. Both the abstract and main text of the retrieved entries were rigorously assessed to guarantee the accuracy of the selection. Two independent investigators screened each record and report.

Studies that met the following eligibility criteria were included in this study: 1) randomized controlled trials (RCTs), 2) patients included in the studies should be diagnosed with GISTs and previously treated with at least two TKIs, and 3) the outcome data should include at least one of PFS, DCR, OS, or AE. Additionally, interim or repetitive reports from the same registered study were excluded. We selected studies with the largest sample sizes and the latest publications.

### Data extraction and assessment of bias risk

General information, efficacy data, and safety data were extracted from the title, abstract, and full text by two different investigators (independently) from our group using pre-designed forms.

The risk of bias of eligible studies was evaluated by two authors based on the Cochrane Risk of Bias tool. In total, seven domains (random sequence generation, allocation concealment, blinding of participants and personnel, blinding of outcome assessment, incomplete outcome data, selective reports, and other bias), and three grades (high, low, and unclear) were assigned. Any incongruity between the interpretations of the authors was resolved by discussion with an independent third party.

### Outcome measures

The primary outcome of this network meta-analysis was PFS, and the secondary outcomes were disease control rate (DCR), OS, and adverse effect (AE). In addition, PFS of subgroups (11 exon mutation, 9 exon mutation, only third-line therapy, and fourth-line and more therapies) was used to perform the subgroup analysis. The hazard ratio (HR) and its 95% confidence interval (CI) that compares the treatments in each study were adopted to calculate the effect size for survival outcomes such as PFS and OS. DCR was defined as the proportion of patients with complete response (CR), partial response (PR), or stable disease (SD) to each treatment. AE was restricted to grades 3–5 AEs presented in each study.

### Statistical analysis

We performed a Bayesian framework network meta-analysis to compare and rank the efficacy and safety of treatments by direct and indirect comparisons, and a random-effects model was used to explain the statistical heterogeneity across the studies with *I* ^2^ statistics and a Bland–Altman test. When *I* ^2^ < 25%, we recognized it as low heterogeneity; however, notable heterogeneity was indicated if *I* ^2^ > 50%. A Markov Chain Monte Carlo (MCMC) method with 25,000 burn-ins and 5000 iterations of 4 for each chain and a thinning interval of one for each outcome was used to obtain the posterior distributions. Model convergence was evaluated using the Brooks–Gelman–Rubin diagnostic plot. The surface under the cumulative ranking (SUCRA) values based on posterior probabilities were used to provide the best option and treatment rankings. Egger’s tests were used to check the publication bias in the network meta-analysis due to a limited quantity of studies. A loop inconsistency test was not available because no loop connection was formed in this study; in that case, we compared degrees of inconsistency (DIC) of the consistency model and inconsistency model to perform the consistency analysis. All processes of the Bayesian network meta-analysis were performed using R software (version 4.1.2; R Foundation for Statistical Computing, Vienna, Austria) combined with JAGS (version 4.3.0).

## Results

### Literature search and study characteristics

**FIGURE 1:**
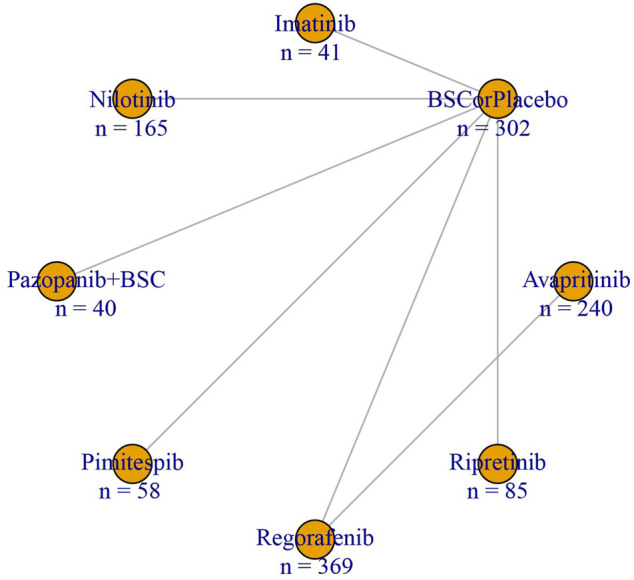
Network structure plot of progress-free survival and the disease control rate for regimens included in the network meta-analysis.

A total of 84 studies were originally retrieved through the literature search. Among them, 71 records remained after removing duplicates; 53 studies were excluded based on titles and abstracts; and 18 studies were selected for the full-text review. Of the potentially eligible full-text studies, seven well-designed and high-quality RCTs containing 1300 patients with GISTs were finally selected for the network meta-analysis (Supplementary Figure S1). The RCTs included in the analysis consisted of 6 phase III trials and 1 phase II trial, which described eight treatment nodes (avapritinib, imatinib, nilotinib, pazopanib + BSC, pimitespib, regorafenib, ripretinib, and best supportive care (BSC) or placebo) (Kang et al., 2021; Mir et al., 2016; Blay et al., 2020; Kurokawa et al., 2022; Demetri et al., 2013; Reichardt et al., 2012; Changhoon and Yoon-Koo, 2015). The network relationship is depicted in Figure 1 and Supplementary Figure S2. We noticed that the number of male participants (63.6%) was greater than female participants (36.4%). The primary outcomes of all RCTs were PFS or OS; in this case, we have rather complete records of PFS data, which is the primary outcome of this network meta-analysis. As for the DCR, which was not a primary outcome of any RCT, we calculated it based on CR, PR, and SD data. All RCTs used BSC or a placebo as a control, except for the study by Kang et al. (2021) (avapritinib vs. regorafenib). Other detailed general characteristics and efficacy and safety data of all RCTs included for the analysis are presented in Tables 1, 2. Furthermore, data extracted for the subgroup analysis are presented in Supplementary Table S1 and S2.

**TABLE 1:** Baseline characteristics of eligible studies in systematic review.

Study	Region	Registration	Phase	Treatment	Control	Sample size (treatment/control)	End point	Previous therapy	Journal
Demetri et al. (2013)	Multicentre	NCT01271712	3	Regorafenib	Placebo	133/66	PFS/DCR/AE	Two or more	Lancet
Mir et al. (2016)	Multicentre	NCT01323400	2	Pazopanib+BSC	BSC	40/41	PFS/DCR/OS	Two or more	Lancet
Reichardt et al. (2012)	Multicentre	NCT00471328	3	Nilotinib	BSC+/-TKI	165/83	PFS/DCR/OS/AE	Two or more	Annals of oncology
Kang et al. (2021)	Multicentre	NCT03465722	3	Avapritinib	Regorafenib	240/236	PFS/DCR/AE	Two or more	Journal of clinical oncology
Kang et al. (2015)	Korea	NCT01151852	3	Imatinib	Placebo	41/40	PFS/DCR/OS/AE	Two or more	Clinical investigation
Blay et al. (2020)	Multicentre	NCT03353753	3	Ripretinib	Placebo	85/44	PFS/DCR/OS	Three or more	Lancet
Kurokawa et al. (2022)	Japan	NA	3	Pimitespib	Placebo	58/28	PFS/DCR/AE	Three or more	NA

Study	Treatment	Median age	Age range	Sample size	Sex (M/F)	ECOG status (0/1 or 2)	Previous TKI
Demetri et al. (2013)	Regorafenib	60	18∼82	133	85/48	73/60	2 or more
Demetri et al. (2013)	Placebo	61	25∼87	66	42/24	37/29	2 or more
Mir et al. (2016)	Pazopanib+BSC	65	33∼85	40	25/15	15/20	2 or more
Mir et al. (2016)	BSC	59	27∼81	41	32/9	12/21	2 or more
Reichardt et al. (2012)	Nilotinib	NA	18∼83	165	101/64	NA	2 or more
Reichardt et al. (2012)	BSC+/-TKI	NA	37∼82	83	47/36	NA	2 or more
Kang et al. (2021)	Avapritinib	61	31∼91	240	162/78	125/115	2 or more
Kang et al. (2021)	Regorafenib	62	31∼86	236	156/80	103/133	2 or more
Kang et al. (2015)	Imatinib	57	52∼65	41	29/12	NA	2 or more
Kang et al. (2015)	Placebo	61	54∼67	40	26/14	NA	2 or more
Blay et al. (2020)	Ripretinib	59	29∼82	85	47/38	37/48	3 or more
Blay et al. (2020)	Placebo	65	33∼83	44	26/18	17/27	3 or more
Kurokawa et al. (2022)	Pimitespib	62	32∼83	58	34/24	49/9	3 or more
Kurokawa et al. (2022)	Placebo	61.5	26∼81	28	15/13	24/4	3 or more

Study	Treatment	Previous systematic anticancer therapy	Duration of previous Imatinib therapy	Disease status at inclusion (metastasis/non-metastasis)	Disease status at diagnosis (metastasis/non-metastasis)	Previous surgery performed	Reason for discontinuation of the last targeted therapy
Demetri et al. (2013)	Regorafenib	74 (n=2); 59 (n>2)	18(<=6 months); 26(6∼18 months); 89(>18 months)	NA	NA	NA	NA
Demetri et al. (2013)	Placebo	39 (n=2); 27 (n>2)	4(<= 6 months); 7(6∼18 months); 55(>18 months)	NA	NA	NA	NA
Mir et al. (2016)	Pazopanib+BSC	23 (n>2)	NA	39/1	19/21	35 (88%)	35 (Progression); 4 (Intolerance); 1 (Other)
Mir et al. (2016)	BSC	21 (n>2)	NA	38/3	19/22	32 (78%)	36 (Progression); 2 (Intolerance); 3 (Other)
Reichardt et al. (2012)	Nilotinib	NA	15(<6 months); 14(6∼12 months); 37(12∼24 months); 37(24∼36 months); 35(36∼48 months); 16(48∼60 months); 11(>=60 months)	NA	NA	NA	NA
Reichardt et al. (2012)	BSC+/-TKI	NA	5(<6 months); 9(6∼12 months); 24(12∼24 months); 19(24∼36 months); 12(36∼48 months); 9(48∼60 months); 5(>=60 months)	NA	NA	NA	NA
Kang et al. (2021)	Avapritinib	207 (n=2); 33 (n=3)	NA	238/2	NA	213 (88.8%)	NA
Kang et al. (2021)	Regorafenib	201 (n=2); 35 (n=3)	NA	231/5	NA	208 (88.1%)	NA
Kang et al. (2015)	Imatinib	16 (n>2)	3(6∼12 months); 14(12∼24 months); 24(>24 months)	NA	NA	NA	NA
Kang et al. (2015)	Placebo	16 (n>2)	5(6∼12 months); 10(12∼24 months); 25(>24 months)	NA	NA	NA	NA
Blay et al. (2020)	Ripretinib	54 (n=3); 31 (n>3)	NA	NA	NA	NA	NA
Blay et al. (2020)	Placebo	27 (n=3); 17 (n>3)	NA	NA	NA	NA	NA
Kurokawa et al. (2022)	Pimitespib	40 (n=3); 18 (n>3)	NA	NA	NA	NA	NA
Kurokawa et al. (2022)	Placebo	15 (n=3); 13 (n>3)	NA	NA	NA	NA	NA

**TABLE 2:** Efficacy and safety data of included studies.

Study	Treatment	Median PFS (95%CI)	PFS HR (95%CI)	Median OS (95%CI)	OS HR (95%CI)	Complete Response (response/sample size)	Partial Response (response/sample size)	Stable Disease (response/sample size)	Disease Controlled Rate (response/ sample size)	G3-5 Adverse Effect (response/sample size)
Demetri et al. (2013)	Regorafenib	4.8 months (4.1∼5.8)	0.27 (0.19∼0.39)	NA	0.77 (0.42∼1.41)	0/133	6/133	95/133	101/133	79/133
Demetri et al. (2013)	Placebo	0.9 months (0.9∼1.1)		NA		0/66	1/66	22/66	23/66	6/66
Mir et al. (2016)	Pazopanib+BSC	3.4 months (2.4∼5.6)	0.59 (0.37∼0.96)	17.8 months (8.4∼21.9)	NA	0/40	0/40	32/40	32/40	NA
Mir et al. (2016)	BSC	2.3 months (2.1∼3.3)		12.9 months (8.4∼20.5)		0/41	1/41	29/41	30/41	NA
Reichardt et al. (2012)	Nilotinib	3.63 months (2.0∼3.8)	0.9 (0.65∼1.26)	12.03 months (8.8∼14.2)	0.79 (0.52∼1.22)	0/165	1/165	86/165	87/165	29/165
Reichardt et al. (2012)	BSC+/-TKI	3.7 months (2.0∼3.9)		10 months (8.2∼12.9)		0/83	0/83	37/83	37/83	10/83
Kang et al. (2021)	Avapritinib	4.2 months (3.7∼5.6)	1.25 (0.99∼1.57)	8.5 months (NA)	NA	0/240	41/240	113/240	154/240	132/240
Kang et al. (2021)	Regorafenib	5.6 months (3.8∼7.2)		9.6 months (NA)		0/236	17/236	159/236	176/236	135/236
Kang et al. (2015)	Imatinib	1.8 months (1.7∼3.6)	0.48 (0.28∼0.82)	8.2 months (5.5∼12.8)	1 (0.58∼1.83)	0/41	0/41	17/41	17/41	20/41
Kang et al. (2015)	Placebo	0.9 months (0.9∼1.7)		7.5 months (4.4∼12.4)		0/40	0/40	6/40	6/40	7/40
Blay et al. (2020)	Ripretinib	6.3 months (4.6∼6.9)	0.15 (0.09∼0.25)	15.1 months (12.3∼15.1)	0.36 (0.21∼0.62)	0/85	8/85	40/85	48/85	NA
Blay et al. (2020)	Placebo	1 month (0.9∼1.7)		6.6 months (4.1∼11.6)		0/44	0/44	2/44	2/44	NA
Kurokawa et al. (2022)	Pimitespib	2.8 months (1.6∼2.9)	0.51 (0.30∼0.87)	13.8 months (9.2∼NA)	0.42 (0.21∼0.85)	0/58	0/58	36/58	36/58	25/58
Kurokawa et al. (2022)	Placebo	1.4 months (0.9∼1.8)		7.6 months (5.3∼14.9)		0/28	0/28	10/28	10/28	8/28

### Bias assessment

The included studies had a low risk of bias because 73.47% of the assessed parameters were scored as low risk. However, unclear risk and high risk were determined as 20.41% and 6.12%, respectively (Supplementary Figure S3).

Specifically, 57% (*n* = 4) and 43% (*n* = 3) of studies were evaluated to have low risk of bias regarding the random sequence generation and allocation concealment, respectively, whereas no high risk of bias was reported in these two core domains. In terms of blinding of participants and personnel, three included trials were assessed and found to have high risk of bias because of open-label design. Because most studies assumed an independent response in reviewing, more than half were determined as having a low risk of bias for blinding of the outcome assessment. We recognized that all trials reported enough outcomes so that all seven studies were determined to have a low risk of bias in terms of incomplete outcome data and selective reporting. Moreover, only one RCT (Reichardt et al., 2012) was determined to have unclear risk because its results for PFS that was evaluated by local investigators did not match well with those of the central review (Supplementary Table S3).

### Network meta-analysis

**FIGURE 2:**
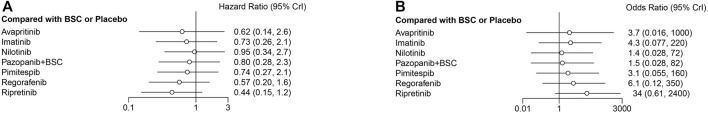
Forest plot of the network meta-analysis for PFS and DCR compared with BSC or the placebo.**(A)** Progression-free survival; **(B)** disease control rate.

**FIGURE 3:**
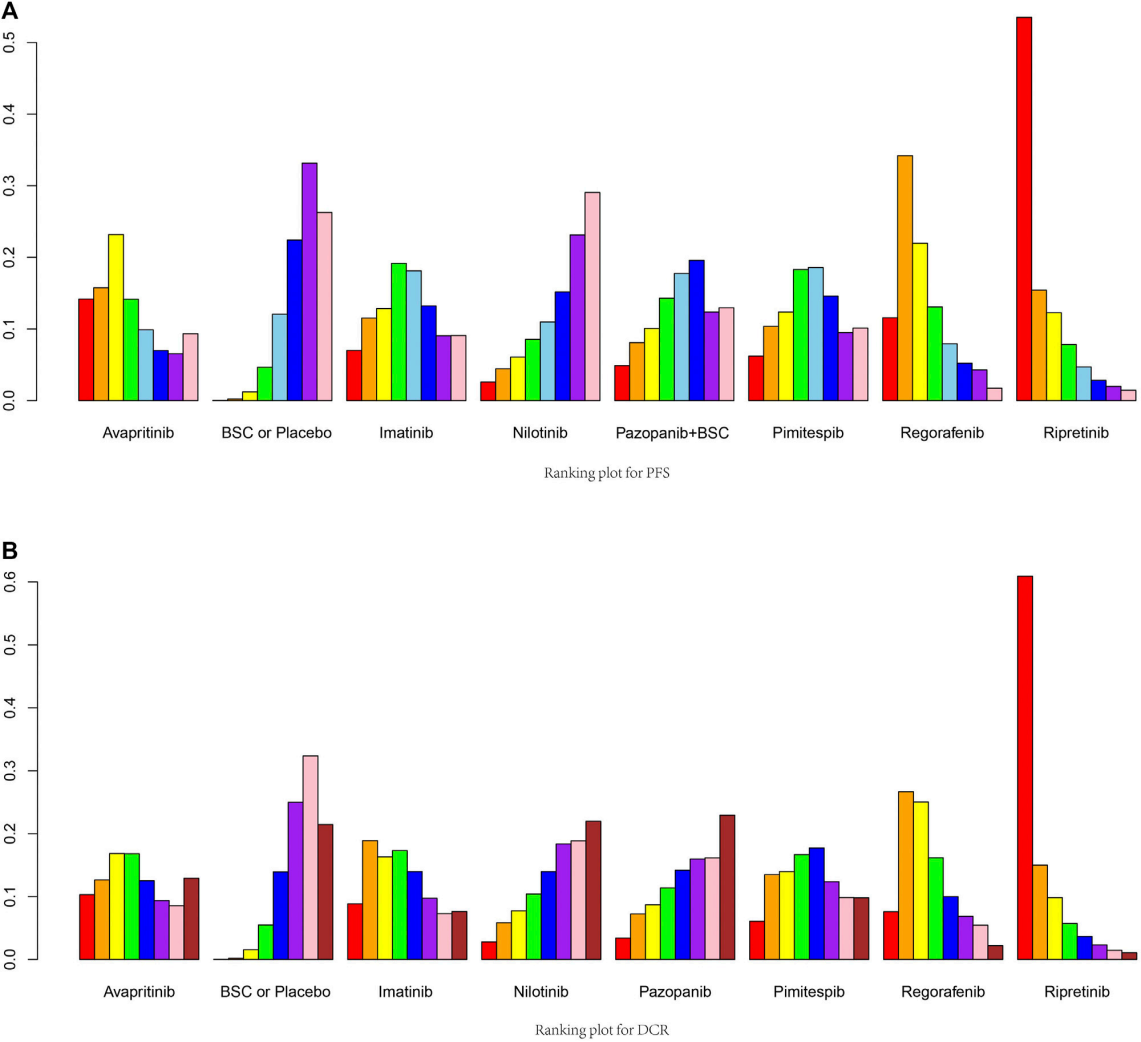
Ranking plot for PFS and DCR. **(A)** Ranking plot for PFS. **(B)** Ranking plot for DCR.

**FIGURE 4:**
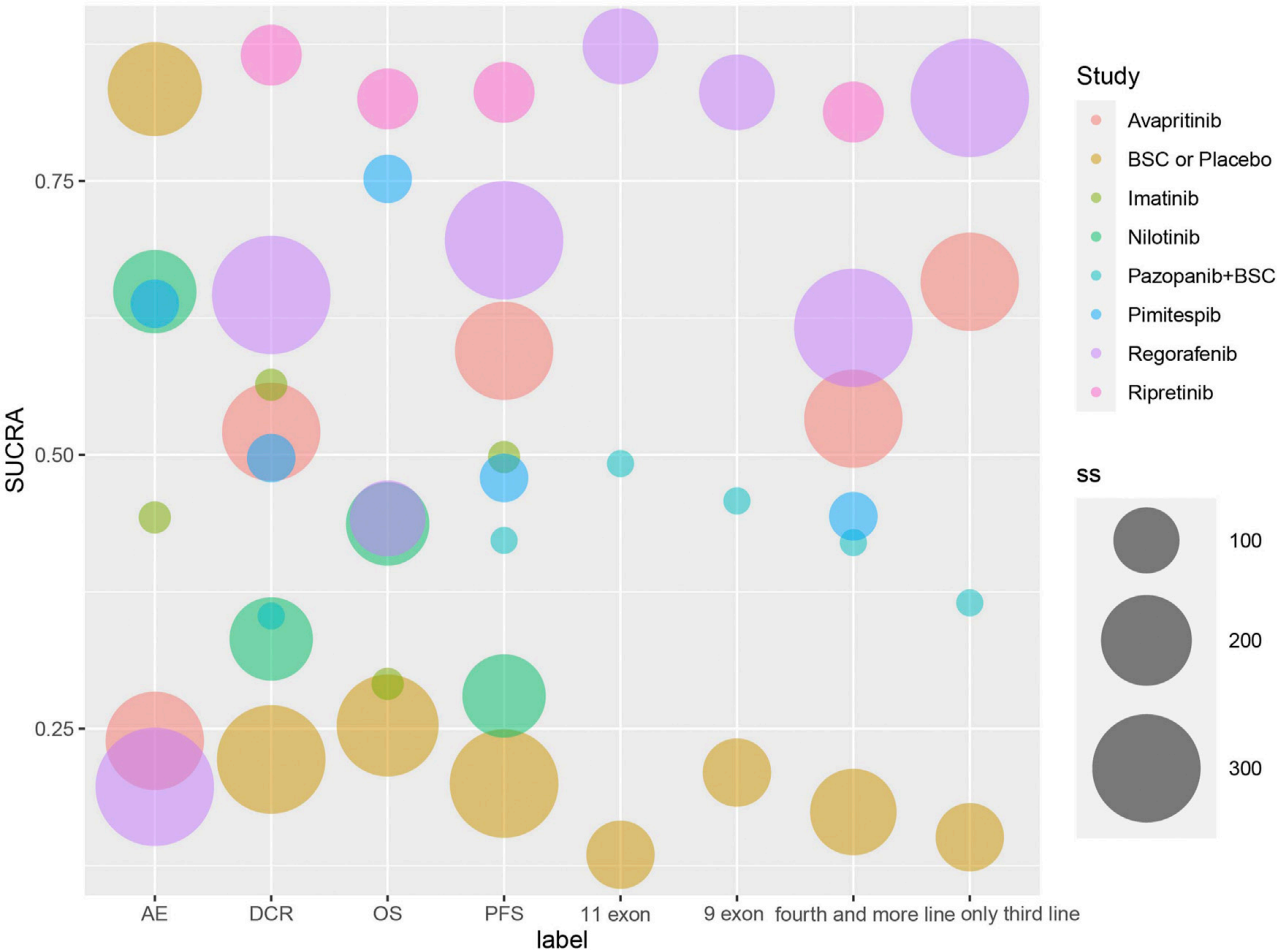
SUCRA score of each targeted agent in all outcomes. The sizes of each circle are weighted by the patient number.

In the Bayesian network meta-analysis, we compared eight treatments in seven eligible trials. The results of the network meta-analysis are shown as a league table for all direct and indirect comparisons (Figures 2, 3; Table 3). We ranked the comparative effects of all agents using SUCRA statistics (Supplementary Table S4). Ripretinib was the optimal choice for improving PFS (83.1%), followed by regorafenib (69.6%) and avapritinib (59.5%; Figure 4). An Egger’s test was used to evaluate publication bias, and a slight publication bias occurred (*p*-value = 0.0655).

**TABLE 3:** Results of the network meta-analysis for PFS.

Ripretinib
0.77 (0.18∼3.4)	Regorafenib						
0.71 (0.12∼4.3)	0.91 (0.33∼2.5)	Avapritinib					
0.61 (0.14∼2.6)	0.78 (0.18∼3.4)	0.86 (0.14∼5.2)	Imatinib				
0.59 (0.13∼2.5)	0.76 (0.18∼3.3)	0.84 (0.14∼4.8)	0.98 (0.22∼4.2)	Pimitespib			
0.55 (0.13∼2.3)	0.71 (0.17∼3.1)	0.78 (0.13∼4.6)	0.91 (0.21∼4.0)	0.93 (0.21∼4.1)	Pazopanib+BSC		
0.46 (0.11∼2.0)	0.59 (0.14∼2.6)	0.65 (0.11∼3.9)	0.76 (0.17∼3.3)	0.78 (0.18∼3.5)	0.84 (0.19∼3.6)	Nilotinib	
0.44 (0.15∼1.2)	0.57 (0.2∼1.6)	0.62 (0.14∼2.6)	0.73 (0.26∼2.1)	0.74 (0.27∼2.1)	0.8 (0.28∼2.3)	0.95 (0.34∼2.7)	BSC or Placebo

With regard to DCR outcomes, all eight treatments were included in the network meta-analysis, which was the same as the PFS outcomes. A league table of all pairwise comparisons is presented in Table 4. Higher scores of SUCRA statistics indicated better comparative effects of the treatments. Similarly, ripretinib represented the best effect in improving DCR (86.5%), whereas regorafenib (64.6%) and imatinib (56.4%) were in second and third place, respectively (Figure 4). The Egger’s test on the DCR dimension showed obvious publication bias (*p*-value <0.001).

**TABLE 4:** Results of the network meta-analysis for DCR.

Ripretinib
5.6 (0.018∼1800)	Regorafenib						
8.1 (0.032∼2800)	1.4 (0.0059∼420)	Imatinib					
9.5 (0.0099∼10000)	1.6 (0.035∼78)	1.2 (0.0012∼1100)	Avapritinib				
11 (0.043∼3600)	2.0 (0.0077∼560)	1.4 (0.0051∼390)	1.2 (0.0015∼1100)	Pimitespib			
23 (0.081∼7200)	4.1 (0.015∼1200)	2.9 (0.011∼680)	2.5 (0.0029∼2100)	2.0 (0.007∼520)	Pazopanib+BSC		
25 (0.094∼7800)	4.4 (0.02∼1200)	3.1 (0.011∼810)	2.7 (0.0035∼2400)	2.2 (0.0082∼580)	1.1 (0.0041∼300)	Nilotinib	
34 (0.61∼2400)	6.1 (0.12∼350)	4.3 (0.077∼220)	3.7 (0.016∼1000)	3.1 (0.055∼160)	1.5 (0.028∼82)	1.4 (0.028∼72)	BSC or Placebo

For OS and severe AE (grade 3–5) outcomes, six treatments from five eligible trials were selected for direct and indirect comparisons, respectively. Detailed results are available in Supplementary Table S5. SUCRA statistics are used for ranking the effects of treatments using both OS and severe AE outcomes. Ripretinib also has the highest probability of performing better than other therapies for improving OS with the highest SUCRA statistics (82.4%). In the other aspects for concomitant milder AEs, BSC used as a control seemed to have the highest tolerance (83.4%) followed by nilotinib (64.9%) and pimitespib (63.8%; Supplementary Table S4). Forest plots of comparisons are located in Supplementary Figure S4. All diagnostics of the models can be visualized in Supplementary Figure S5 and S6. Rank plots are exhibited in Supplementary Figure S7. The grade 3–5 AEs of pazopanib and ripretinib treatments were not included for comparison because of the inconsistent data format.

Additionally, we extracted data to form four subgroups consisting of outcomes of 9 exon mutation PFS (three treatment nodes with two eligible studies), 11 exon mutation PFS (three treatment nodes with two eligible studies), only third-line therapy PFS (four treatment nodes with three eligible studies), and fourth-line and more therapies PFS (six treatment nodes with five eligible studies). Limited studies consisted of PFS data specifically for 9 exon mutations and 11 exon mutations, and the analysis results of 9 exon mutations and 11 exon mutations were similar (regorafenib preformed the best with SUCRA statistics of 87.3% and 83.1%, respectively). For the third-line therapy subgroup only, regorafenib was also the optimal choice statistically (SUCRA statistics: 82.6%) followed by avapritinib (SUCRA statistics: 65.8%). However, ripretinib had the most favorable effect comparably (SUCRA statistics: 81.3%) in the subgroup of patients who received fourth-line or beyond therapies. Detailed information is available in Supplementary Table S4 (SUCRA statistics table) and S5 (League table).

### Heterogeneity and inconsistency tests

Based on *I* ^2^ statistics and Bland–Altman test plots (Supplementary Figure S8), no notable heterogeneity was observed. All eligible studies included in this Bayesian network meta-analysis are BSC controlled, except for one study that selected the regorafenib intervention as the control group. For this reason, no loop connection was formed in the network relationship construction process, and no global *I* ^2^ test or loop inconsistency tests were available. Instead, we compared DICs of the consistency model and inconsistency model for each outcome. All differences were <1, which implied that this network meta-analysis was well fitted with the consistency hypothesis.

## Discussion

To our knowledge, this is the first Bayesian network meta-analysis to assess the comparative efficacy and tolerance of third-line or over third-line therapies against GIST. Zhang et al. performed a Bayesian network meta-analysis on targeted therapies after the prior failure of TKIs (Zhang et al., 2021). However, in this study, we aimed at third-line and over third-line therapies and analyzed more beneficial clinical outcomes using random effects models.

In the network meta-analysis of seven RCTs consisting of a total of 1300 patients with refractory GIST, we simultaneously analyzed eight treatment nodes including seven TKIs and one placebo/BSC reference. We found that ripretinib was superior to other regimens with respect to both PFS and DCR, which displayed higher SUCRA values, especially in the fourth-line therapy subgroup. It was also determined that pimitespib and nilotinib had better tolerance.

This indicated that imatinib could achieve an overall response rate (ORR) of >50% (CR: 5%; PR: 48.3%) (Demetri et al., 2002; van Oosterom et al., 2002; Verweij et al., 2004; Kubota, 2006). Nevertheless, none of the eligible trials for this systematic review reached CRs, and only a few PRs were reported. The ORR in several studies might be 0, which had comparatively no significance. For this reason, we selected DCR instead of ORR as an outcome to evaluate the efficacy of the treatments.

Ripretinib, a broad-spectrum KIT and PDGFRA switch-control TKI, was the most effective agent in improving PFS, OS, and DCR (Dhillon, 2020). The heterogeneity of drug-resistant KIT mutations has been a major challenge in the treatment of GISTs; the presence of a broad inhibitor with outstanding effectiveness is inspiring (Smith et al., 2019).

Ripretinib is approved for the treatment of adult patients with advanced GIST who have received prior treatment with ≥3 TKIs including imatinib (Lostes-Bardaji et al., 2021). Recently, a phase III study of ripretinib versus sunitinib for the treatment of advanced GISTs after the failure of imatinib (INTRIGUE) was completed (Bauer et al., 2022). However, ripretinib did not meet the primary end point of superiority in the PFS over sunitinib (8 months vs. 8.3 months), whereas ORR was higher for patients receiving ripretinib in the KIT exon 11 population and ripretinib has a more favorable safety profile (Bauer et al., 2022). Although ripretinib did not seem to defeat sunitinib regarding second-line therapy for GISTs, it still performed better in this network meta-analysis, which suggests ripretinib as a high priority in fourth-line therapy.

More specifically, regorafenib was best in improving PFS in the subgroup analysis of only third-line therapy. Regorafenib is a multi-target inhibitor for KIT, platelet-derived growth factor receptors (PDGFRs), VEGFR, fibroblast growth factor receptors, RET, and BRAF and is suggested for the third-line therapy for GISTs based on the guidelines (Nishida et al., 2016). The direct comparative trial VOYAGER (Kang et al., 2021) (avapritinib vs. regorafenib) also recently failed. Although traditional TKIs, such as sunitinib and regorafenib, did not show an ideal effectiveness as novel TKIs in the trial stage, they are still reliable for clinical administration.

The available research data show that the sensitivity of different genetic subtypes to drugs is inconsistent in GIST. Clinical activity of sunitinib after imatinib failure is significantly influenced by both primary and secondary mutations in the predominant pathogenic kinase, which has implications for optimization of the treatment of patients with GISTs, and regorafenib has also shown variable sensitivity to multiple resistant tumor cell clones driven by additional mutations in KIT or PDGFRA (Heinrich et al., 2008; Yeh et al., 2017; Blay et al., 2021). Avapritinib has excellent efficacy in the treatment of unresectable or metastatic PDGFRA D842V mutant GISTs (Heinrich et al., 2020). Ripretinib, as a broad-spectrum KIT and PDGFRA switch-control TKI, showed excellent efficacy against various primary and secondary mutation types in the fourth-line treatment of GIST, although it can be observed from the genotyping data of the INTRIGUE study that efficacy against different genetic subtypes is still different (Blay et al., 2020; Bauer et al., 2022). Precise targeted therapy is a very important direction for the future treatment of GIST, and more high-quality studies of new agents are expected. We analyzed the PFS outcome of the 11-exon mutation and 9 exon mutation subgroups. The results of a few studies consisted of survival status correlated with genomic analysis, and only 2 studies with 3 treatment nodes were included in the network meta-analysis. This limited sample size might reduce the persuasiveness of the comparison. However, along with the unsatisfying results of the INTRIGUE trial, which temporarily denied an all different mutation types, the next breakthrough of a new drug should focus on specific gene types. In this case, we believe that more gene-specific prognostic survival data would be particularly emphasized in the coming clinical trials. In our vision, gene-mutation-specific survival is instructive.

In this network meta-analysis, we noticed a new drug (pimitespib, TAS-116) administered for the treatment of GISTs (Kurokawa et al., 2022). Pimitespib is an oral heat shock protein 90 (HSP90) inhibitor that is used for the treatment of T-cell leukemia or colorectal cancer (Kawazoe et al., 2021; Ikebe et al., 2022). Pimitespib also has the potential of improving OS and shows a high tolerance in our review.

This study has some limitations.

First, this study was limited to estimations that were based on the data availability. As the assessment of OS required a long period of time, Mir et al. (2016) and Kang et al. (2021) did not report the HR of OS because it was not reached. In terms of AEs, the total number of grade 3–5 AEs were not present in studies by Mir et al. (2016) and Blay et al. (2020), and we could not calculate it by the accumulation either because patients might suffer from more than one pattern of AE; therefore, pazopanib and ripretinib were not included in the comparison even though ripretinib was reported to have a more favorable safety profile compared with sunitinib (Bauer et al., 2022).

Second, because of the low number of included studies and each comparison between treatments consisted of only one study, the statistical power might be relatively lower in the case of heterogeneity. However, no obvious heterogeneity was found in the present meta-analysis.

Third, this network meta-analysis was performed based on the summary or aggregated data instead of individual data, which is common in meta-analyses. However, we did not have access to enough detailed data for the subgroup analysis.

Fourth, publication bias actually did exist in the present network meta-analysis. It is natural that positive outcomes indicating novel and effective drugs are more likely to be published. A few clinical trials on new drugs which were non-effective in a prior stage could be withdrawn. Additionally, RCTs focused on GISTs were limited, which could be another source of publication bias contributing to the rarity.

Fifth, no loop structure was constructed in the network relationship plot. For this reason, we did not perform a loop inconsistency test to assess the inconsistencies in this network meta-analysis. We hope that more direct comparisons of the agents can be carried out.

Sixth, though combined as treatments for refractory GIST, the third-line and fourth-line therapies could still be under different clinical contexts. We performed a subgroup analysis to represent detailed results after the comparison.

## Conclusion

We verified that regorafenib and ripretinib could be more reliable regimens for third-line and over-third-line therapies, respectively. In pooled analysis, ripretinib expressed priority in improving PFS, OS, and DCR. Both nilotinib and pimitespib presented favorable tolerance, whereas their efficacy still needs to be verified. Pimitespib is a novel potential agent for the treatment of GISTs, and more details are expected. Using an inhibitor for a specific gene mutation type may be a new trend in the future. We hope that more high-quality studies of new agents can bring new information to light in the area of precise targeted therapy for GISTs.

## Data Availability

The original contributions presented in the study are included in the article/Supplementary Material, and further inquiries can be directed to the corresponding author.
