# Supplementary figures and images for: A systematic review and network meta-analysis of the efficacy and safety of third-line and over third-line therapy after imatinib and TKI resistance in advanced gastrointestinal stromal tumor
Source: Front Pharmacol. 2022 Nov 21;13:978885. doi: 10.3389/fphar.2022.978885 (PMC9720279; doi:10.3389/fphar.2022.978885)

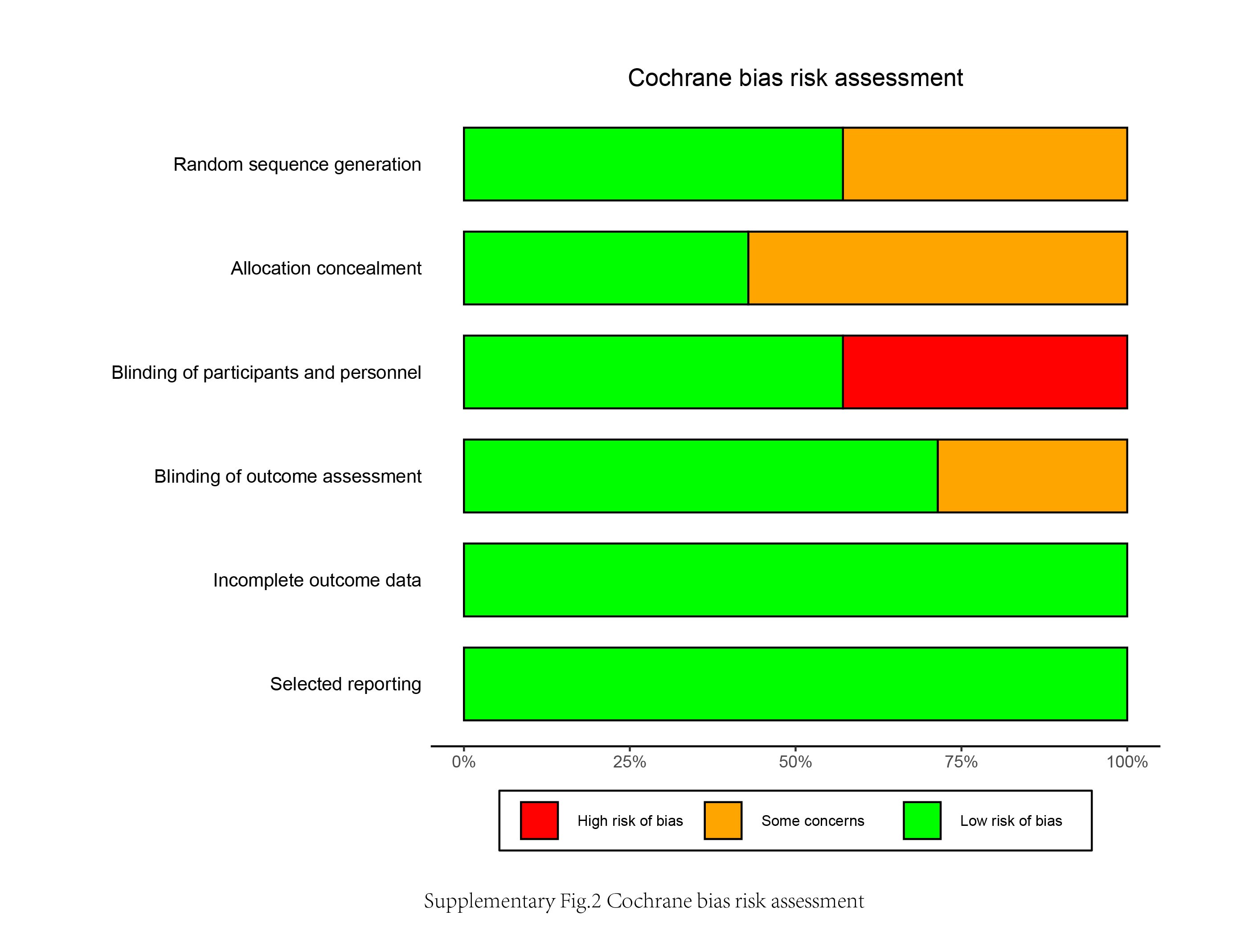

Supplement: Supplementary file 1 [file Image3.JPEG]

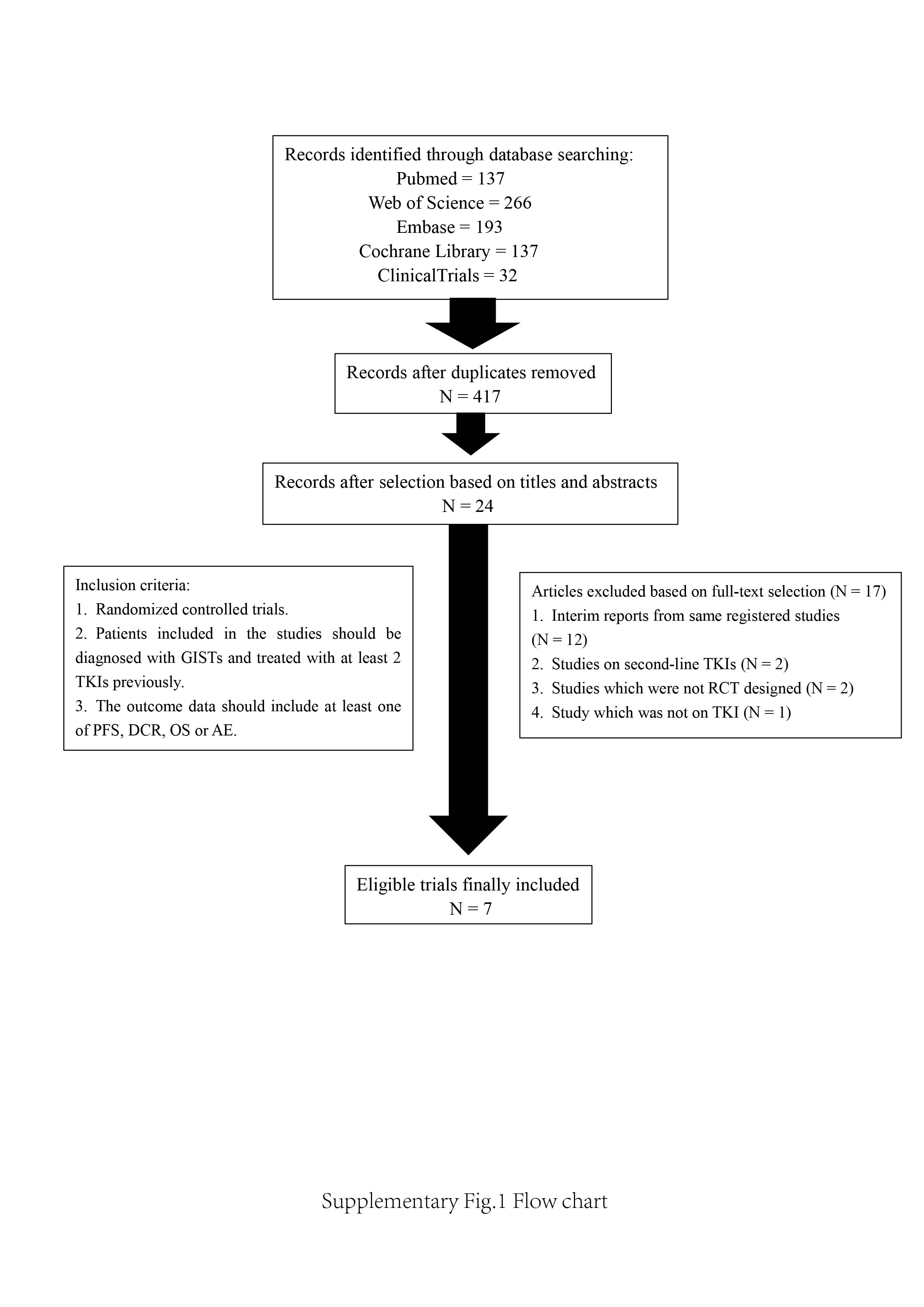

Supplement: Supplementary file 3 [file Image1.JPEG]

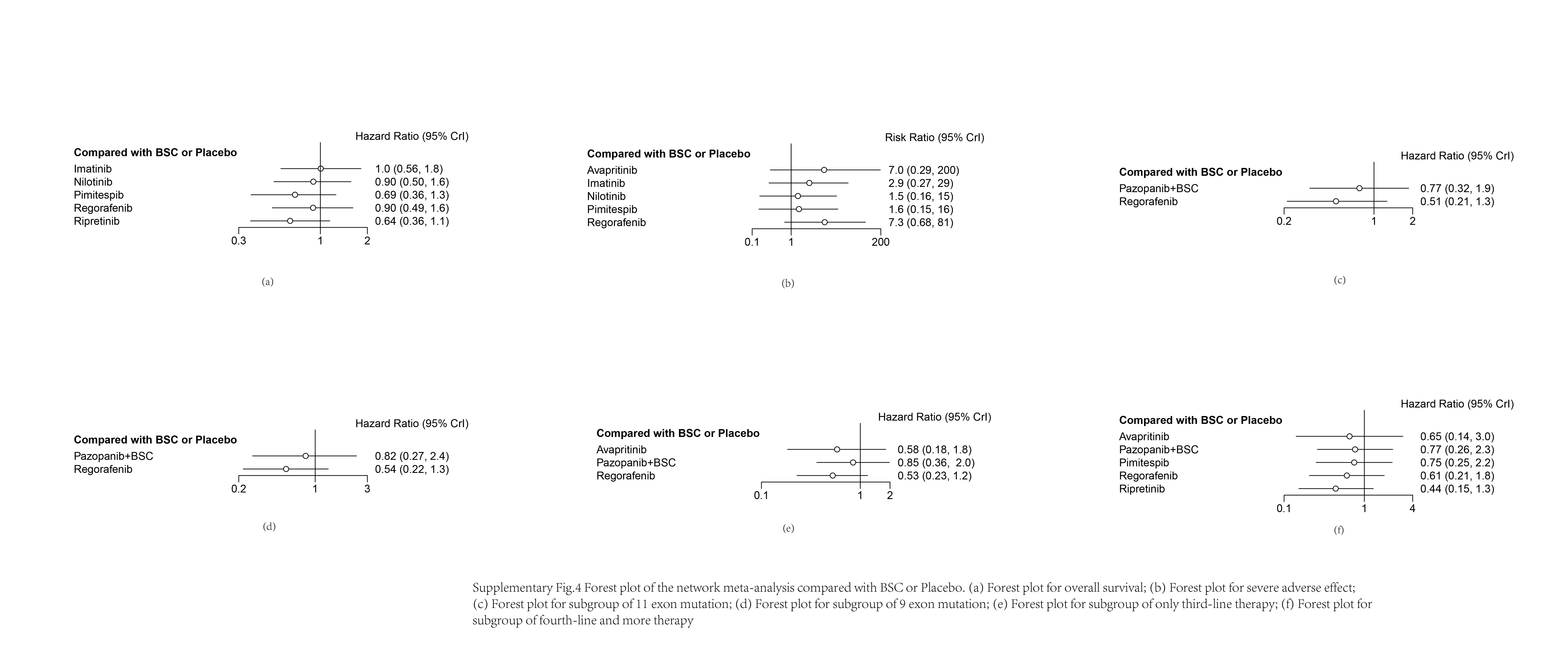

Supplement: Supplementary file 4 [file Image4.JPEG]

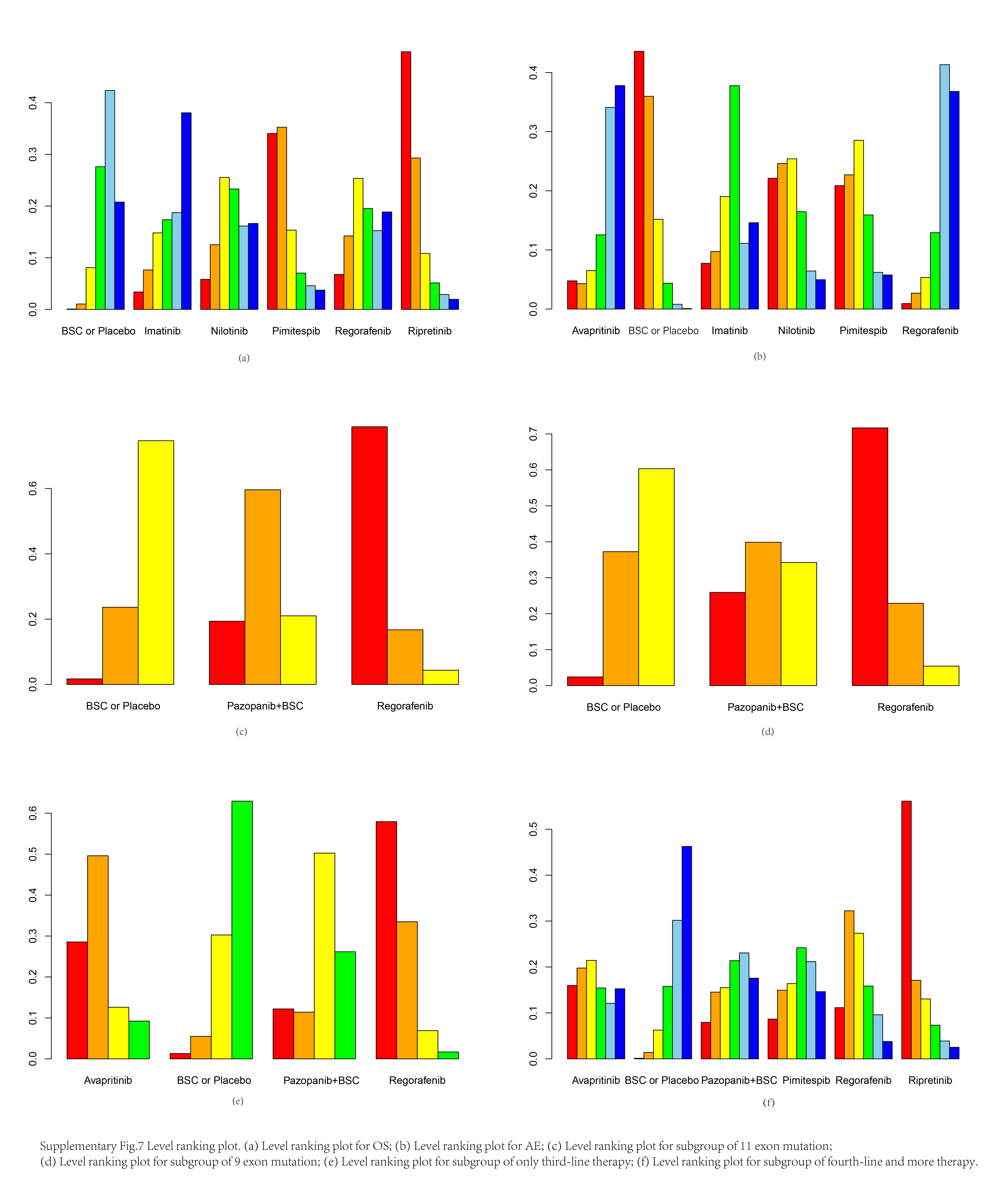

Supplement: Supplementary file 5 [file Image7.JPEG]

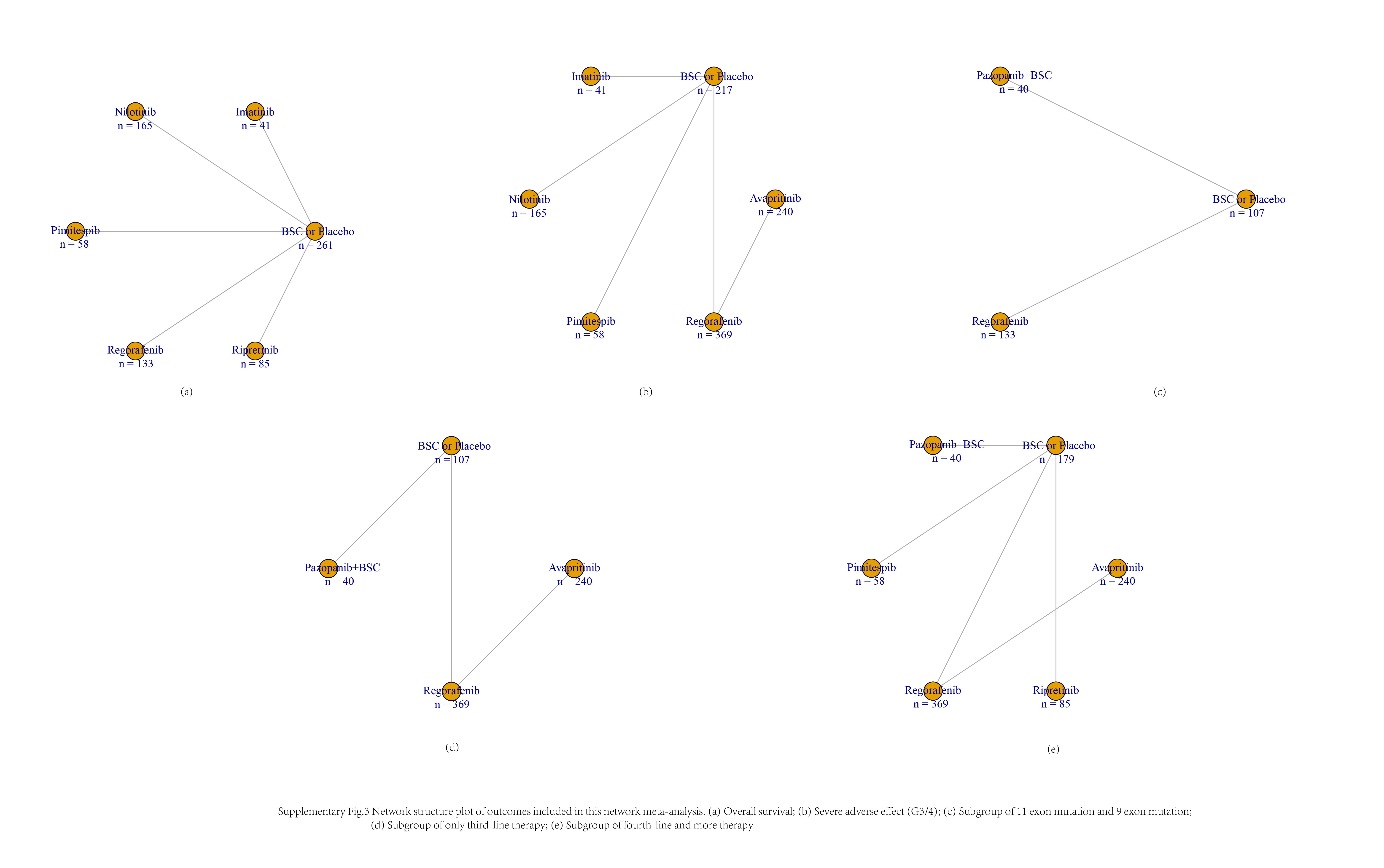

Supplement: Supplementary file 6 [file Image2.JPEG]

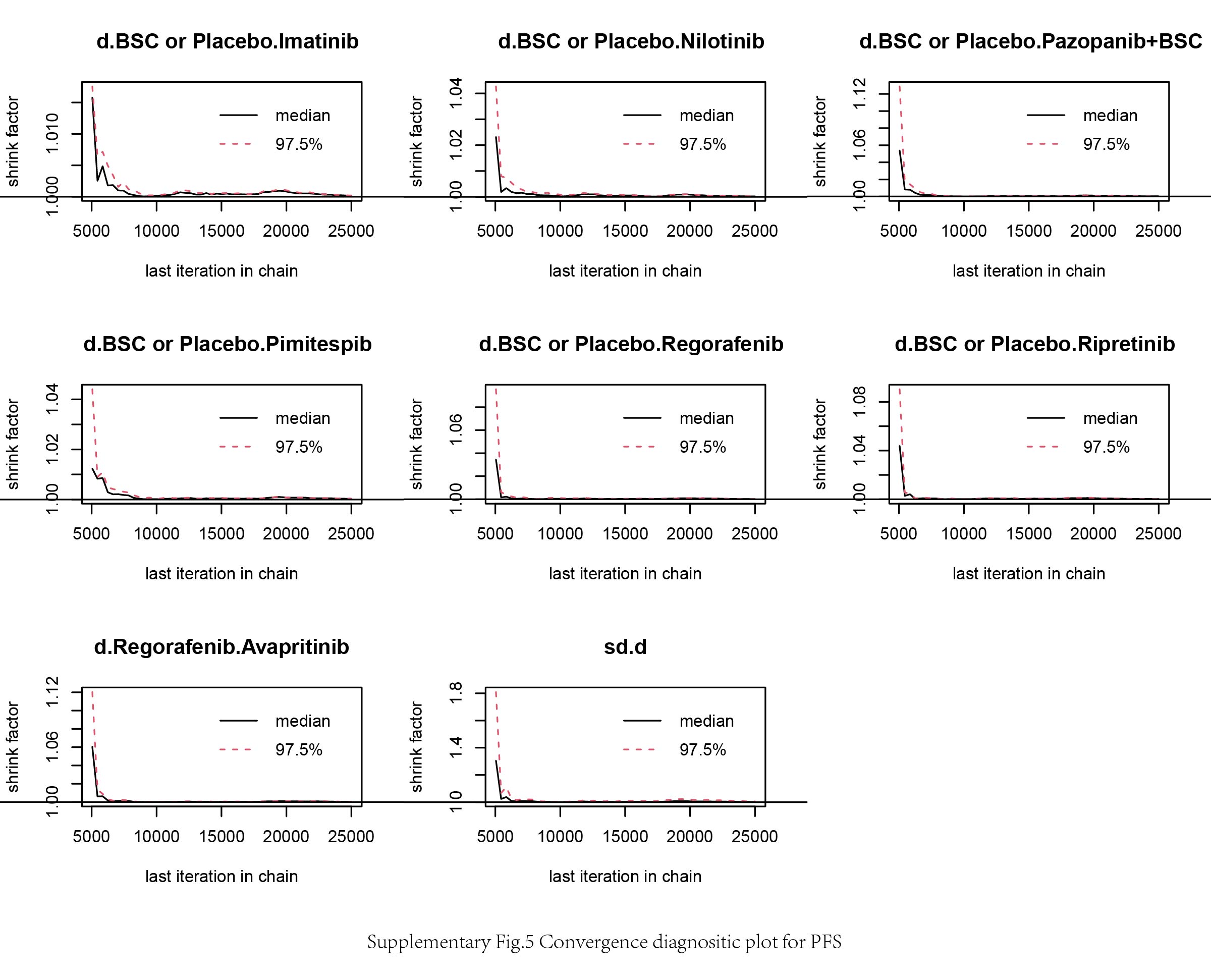

Supplement: Supplementary file 7 [file Image5.JPEG]

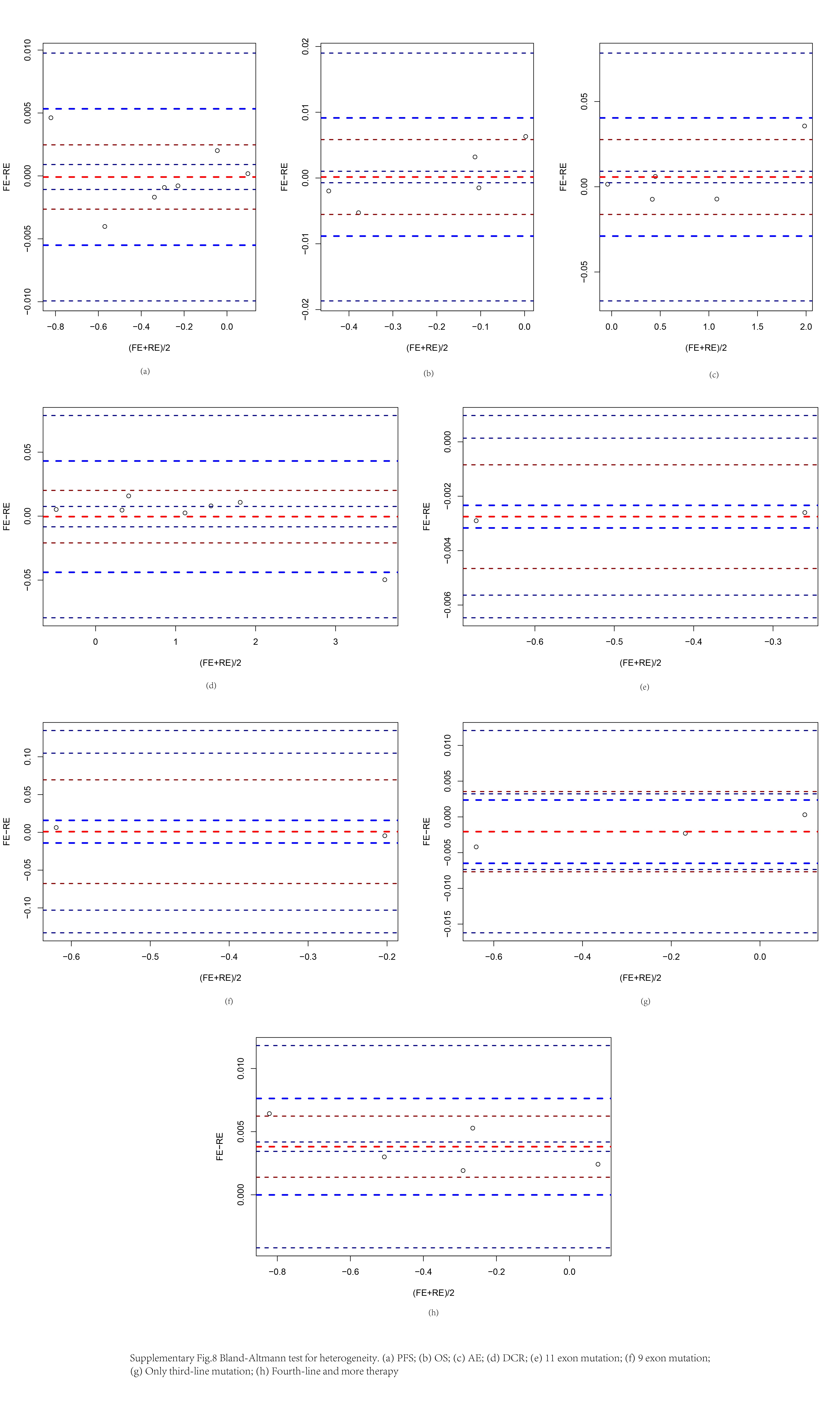

Supplement: Supplementary file 13 [file Image8.JPEG]

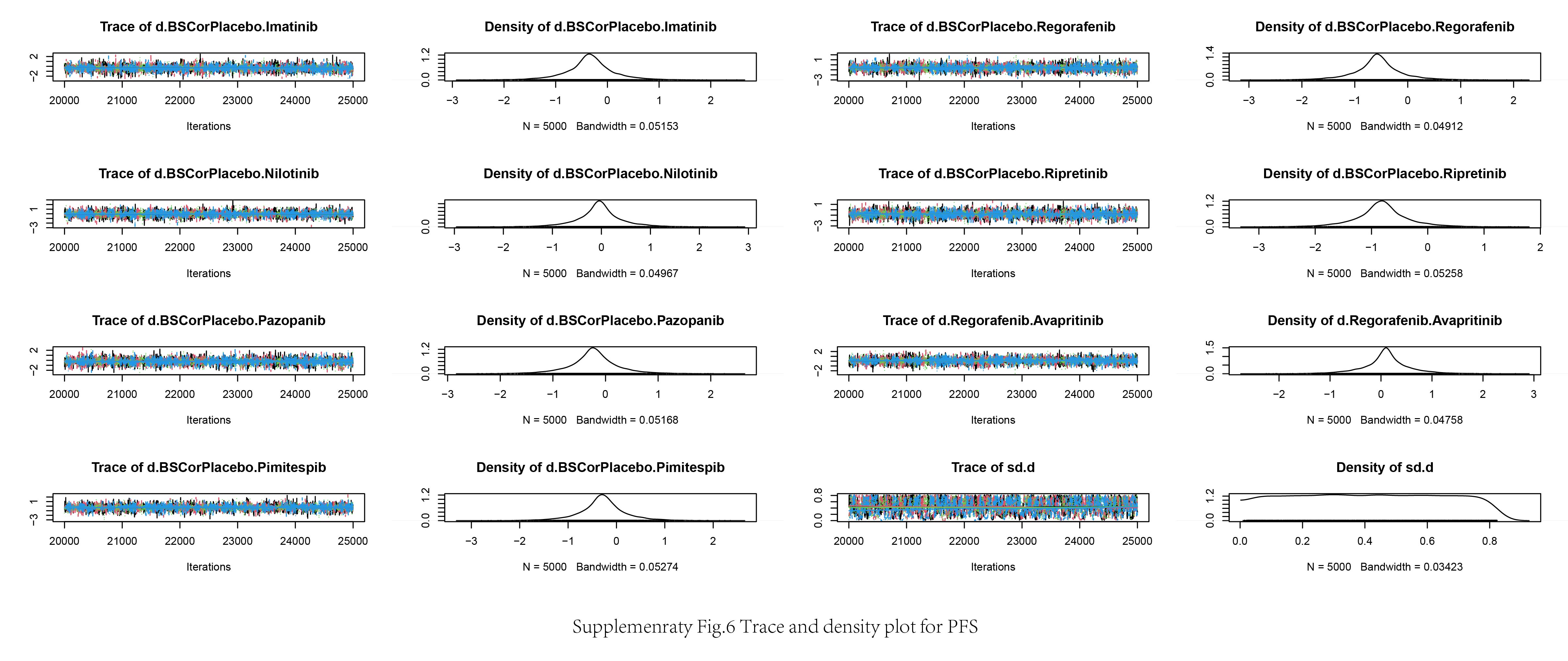

Supplement: Supplementary file 14 [file Image6.JPEG]
