# Supplementary material for: A systematic review and network meta-analysis of the efficacy and safety of third-line and over third-line therapy after imatinib and TKI resistance in advanced gastrointestinal stromal tumor
Source: Front Pharmacol. 2022 Nov 21;13:978885. doi: 10.3389/fphar.2022.978885 (PMC9720279; doi:10.3389/fphar.2022.978885)
Supplement: Supplementary file 2 [file Table1.docx]

**Supplementary Table.1 Efficacy and safety data of included studies for subgroup analysis**

| Study | Exon 11 PFS HR | Exon 11 PFS HR 95% CI | Exon 9 PFS HR | Exon 9 PFS HR 95% CI | True Third Line PFS HR | True Third Line PFS HR 95% CI | Fourth Line and more PFS HR | Fourth Line and more PFS HR 95% CI |
| --- | --- | --- | --- | --- | --- | --- | --- | --- |
| Demetri et al. 2013 | 0.212 | 0.098~0.458 | 0.239 | 0.065~0.876 | 0.23 | 0.14~0.37 | 0.31 | 0.18~0.54 |
| Demetri et al. 2013 | 1 |  | 1 |  | 1 |  | 1 |  |
| Mir et al. 2016 | 0.55 | 0.27~1.11 | 0.62 | 0.1~3.83 | 0.68 | 0.33~1.41 | 0.54 | 0.29~1.03 |
| Mir et al. 2016 | 1 |  | 1 |  | 1 |  | 1 |  |
| Reichardt et al. 2012 | NA | NA | NA | NA | NA | NA | NA | NA |
| Reichardt et al. 2012 | NA |  | NA |  | NA |  | NA |  |
| Kang et al. 2021 | NA | NA | NA | NA | 1.26 | 0.981~1.609 | 1.19 | 0.663~2.149 |
| Kang et al. 2021 | NA |  | NA |  | 1 |  | 1 |  |
| Kang et al. 2015 | NA | NA | NA | NA | NA | NA | NA | NA |
| Kang et al. 2015 | NA |  | NA |  | NA |  | NA |  |
| Blay et al. 2020 | NA | NA | NA | NA | NA | NA | 0.15 | 0.09~0.25 |
| Blay et al. 2020 | NA |  | NA |  | NA |  | 1 |  |
| Kurokawa et al. 2022 | NA | NA | NA | NA | NA | NA | 0.51 | 0.3~0.87 |
| Kurokawa et al. 2022 | NA |  | NA |  | NA |  | 1 |  |
