# Supplementary material for: A systematic review and network meta-analysis of the efficacy and safety of third-line and over third-line therapy after imatinib and TKI resistance in advanced gastrointestinal stromal tumor
Source: Front Pharmacol. 2022 Nov 21;13:978885. doi: 10.3389/fphar.2022.978885 (PMC9720279; doi:10.3389/fphar.2022.978885)
Supplement: Supplementary file 8 [file Table2.docx]

**Supplementary Table.2 Gene mutation situation of population included**

| Study | KIT 11 exon mutation | KIT 9 exon mutation | KIT 13 exon mutation | KIT 17 exon mutation | PDGFRA 12 exon mutation | PDGFRA 14 exon mutation | PDGFRA 18 exon mutation | Wild type | KIT V654A/T670I |
| --- | --- | --- | --- | --- | --- | --- | --- | --- | --- |
| Demetri et al. 2013 | 51 | 15 | NA | NA | NA | NA | NA | NA | NA |
| Mir et al. 2016 | 37 | 6 | 1 | 2 | 1 | 0 | 7 | 6 | NA |
| Reichardt et al. 2012 | NA | NA | NA | NA | NA | NA | NA | NA | NA |
| Kang et al. 2021 | NA | NA | NA | 109 | NA | NA | 18 | NA | 67 |
| Kang et al. 2015 | 62 | 10 | NA | NA | NA | NA | NA | NA | NA |
| Blay et al. 2020 | 75 | 20 | NA | NA | NA | NA | NA | 10 | NA |
| Kurokawa et al. 2022 | 37 | 8 | NA | NA | NA | NA | NA | NA | NA |
