# Supplementary material for: A systematic review and network meta-analysis of the efficacy and safety of third-line and over third-line therapy after imatinib and TKI resistance in advanced gastrointestinal stromal tumor
Source: Front Pharmacol. 2022 Nov 21;13:978885. doi: 10.3389/fphar.2022.978885 (PMC9720279; doi:10.3389/fphar.2022.978885)
Supplement: Supplementary file 9 [file Table5.docx]

**Supplementary Table.5 Results of the network meta-analysis**

Results of the network meta-analysis for OS

| Ripretinib |  |  |  |  |  |
| --- | --- | --- | --- | --- | --- |
| 0.93 (0.40~2.2) | Pimitespib |  |  |  |  |
| 0.72 (0.31~1.7) | 0.76 (0.33~1.8) | Regorafenib |  |  |  |
| 0.71 (0.32~1.6) | 0.76 (0.33~1.7) | 0.99 (0.43~2.3) | Nilotinib |  |  |
| 0.64 (0.27~1.5) | 0.68 (0.29~1.6) | 0.89 (0.38~2.1) | 0.89 (0.39~2.0) | Imatinib |  |
| 0.64 (0.36~1.1) | 0.69 (0.36~1.3) | 0.90 (0.49~1.6) | 0.90 (0.50~1.6) | 1.0 (0.56~1.8) | BSC or Placebo |

Results of the network meta-analysis for G3/G4 AE

| BSC or Placebo |  |  |  |  |  |
| --- | --- | --- | --- | --- | --- |
| 0.67 (0.068~6.4) | Nilotinib |  |  |  |  |
| 0.63 (0.063~6.7) | 0.96 (0.036~26) | Pimitespib |  |  |  |
| 0.34 (0.034~3.7) | 0.51 (0.019~15) | 0.54 (0.02~15) | Imatinib |  |  |
| 0.14 (0.0051~3.4) | 0.21 (0.0037~11) | 0.22 (0.0041~13) | 0.41 (0.0073~23) | Avapritinib |  |
| 0.14 (0.012~1.5) | 0.21 (0.0071~5.3) | 0.21 (0.0074~6.4) | 0.4 (0.014~11) | 0.96 (0.1~8.8) | Regorafenib |

Results of the network meta-analysis for subgroup of 11 exon mutation

| Regorafenib |  |  |
| --- | --- | --- |
| 0.66 (0.19~2.4) | Pazopanib+BSC |  |
| 0.51 (0.21~1.3) | 0.77 (0.77~1.9) | BSC or Placebo |

Results of the network meta-analysis for subgroup of 9 exon mutation

| Regorafenib |  |  |
| --- | --- | --- |
| 0.66 (0.17~2.7) | Pazopanib+BSC |  |
| 0.54 (0.22~1.3) | 0.82 (0.27~2.4) | BSC or Placebo |

Results of the network meta-analysis for subgroup of only third line therapy

| Regorafenib |  |  |  |
| --- | --- | --- | --- |
| 0.9 (0.4~2) | Avapritinib |  |  |
| 0.62 (0.19~2.1) | 0.69 (0.17~2.8) | Pazopanib+BSC |  |
| 0.53 (0.23~1.2) | 0.58 (0.18~1.8) | 0.85 (0.36~2) | BSC or Placebo |

Results of the network meta-analysis for subgroup of only fourth line and more lines therapy

| Ripretinib |  |  |  |  |  |
| --- | --- | --- | --- | --- | --- |
| 0.73 (0.16~3.3) | Regorafenib |  |  |  |  |
| 0.68 (0.11~4.3) | 0.93 (0.31~2.7) | Avapritinib |  |  |  |
| 0.59 (0.13~2.6) | 0.81 (0.18~3.7) | 0.87 (0.13~5.8) | Pimitespib |  |  |
| 0.57 (0.12~2.7) | 0.79 (0.17~3.6) | 0.85 (0.13~5.6) | 0.97 (0.21~4.5) | Pazopanib+BSC |  |
| 0.44 (0.15~1.3) | 0.61 (0.21~1.8) | 0.65 (0.14~3.0) | 0.75 (0.25~2.2) | 0.77 (0.26~2.3) | BSC or Placebo |
