# Supplementary material for: A systematic review and network meta-analysis of the efficacy and safety of third-line and over third-line therapy after imatinib and TKI resistance in advanced gastrointestinal stromal tumor
Source: Front Pharmacol. 2022 Nov 21;13:978885. doi: 10.3389/fphar.2022.978885 (PMC9720279; doi:10.3389/fphar.2022.978885)
Supplement: Supplementary file 10 [file Table3.docx]

**Supplementary Table.3 Risk of bias assessment of eligible studies in systematic reviews**

| **Study** | **Random sequence generation** | **Allocation concealment** | **Blinding of participants and personnel** | **Blinding of outcome assessment** | **Incomplete outcome data** | **Selective reporting** | **Other source of bias** |
| --- | --- | --- | --- | --- | --- | --- | --- |
| **Demetri 2013** | **Low: computer-generated randomized list** | **Low: interactive voice response system** | **Low: double-blind placebo-controlled trial** | **Low: blinded central radiology review was performed** | **Low: outcome data is complete** | **Low: All expected outcomes were included** | **Low: well-balanced baseline features** |
| **Mir 2016** | **Low: Statistician generation** | **Low: interactive web-based centralized registration platform allocation** | **High: open-label design** | **Unclear: no specific description** | **Low: outcome data is complete** | **Low: All expected outcomes were included** | **Low: well-balanced baseline features** |
| **Reichardt 2012** | **Unclear: no specific description** | **Unclear: no specific description** | **High: open-label design** | **Low: each scan was evaluated subsequently by blinded central radiology review** | **Low: outcome data is complete** | **Low: All expected outcomes were included** | **Unclear: Results of PFS by local investigators didn’t match well with that of central review** |
| **Kang 2021** | **Unclear: no specific description** | **Unclear: no specific description** | **High: open-label design** | **Unclear: no specific description** | **Low: outcome data is complete** | **Low: All expected outcomes were included** | **Low: well-balanced baseline features** |
| **Kang 2013** | **Low: random permuted block method** | **Low: interactive response technology** | **Low: double-blind placebo-controlled trial** | **Low: a masked external radiology central review was performed** | **Low: outcome data is complete** | **Low: All expected outcomes were included** | **Low: well-balanced baseline features** |
| **Blay 2020** | **Low: random permuted block method** | **Unclear: no specific description** | **Low: double-blind placebo-controlled trial** | **Low: tumor assessments were done on the basis of blinded independent central review** | **Low: outcome data is complete** | **Low: All expected outcomes were included** | **Low: well-balanced baseline features** |
| **Kurokawa 2022** | **Unclear: no specific description** | **Unclear: no specific description** | **Low: double-blind placebo-controlled trial** | **Low: blinded central radiological review was performed** | **Low: outcome data is complete** | **Low: All expected outcomes were included** | **Low: well-balanced baseline features** |
