# Supplementary material for: A systematic review and network meta-analysis of the efficacy and safety of third-line and over third-line therapy after imatinib and TKI resistance in advanced gastrointestinal stromal tumor
Source: Front Pharmacol. 2022 Nov 21;13:978885. doi: 10.3389/fphar.2022.978885 (PMC9720279; doi:10.3389/fphar.2022.978885)
Supplement: Supplementary file 11 [file Table4.docx]

**Supplementary Table.4 SUCRA table**

|  | Avapritinib | BSC or Placebo | Imatinib | Nilotinib | Pazopanib+BSC | Pimitespib | Regorafenib | Ripretinib |
| --- | --- | --- | --- | --- | --- | --- | --- | --- |
| PFS | 0.595 | 0.200 | 0.498 | 0.280 | 0.422 | 0.479 | 0.696 | 0.831 |
| OS |  | 0.253 | 0.291 | 0.437 |  | 0.752 | 0.442 | 0.825 |
| DCR | 0.521 | 0.222 | 0.564 | 0.332 | 0.353 | 0.497 | 0.646 | 0.865 |
| G3-5 AE | 0.239 | 0.834 | 0.443 | 0.649 |  | 0.638 | 0.197 |  |
| Exon 11 mutation subgroup PFS |  | 0.135 |  |  | 0.492 |  | 0.873 |  |
| Exon 9 mutation subgroup PFS |  | 0.210 |  |  | 0.458 |  | 0.831 |  |
| Only third line therapy subgroup PFS | 0.658 | 0.151 |  |  | 0.365 |  | 0.826 |  |
| Only fourth line and more therapy PFS | 0.533 | 0.174 |  |  | 0.420 | 0.444 | 0.616 | 0.813 |
