# Supplementary material for: A systematic review and network meta-analysis of the efficacy and safety of third-line and over third-line therapy after imatinib and TKI resistance in advanced gastrointestinal stromal tumor
Source: Front Pharmacol. 2022 Nov 21;13:978885. doi: 10.3389/fphar.2022.978885 (PMC9720279; doi:10.3389/fphar.2022.978885)
Supplement: Supplementary file 12 [file Table6.docx]

**Searching strategy**

**Pubmed**

1. **((gastrointestinal stromal tumor) OR (Stromal Tumor, Gastrointestinal) OR (Stromal Tumors, Gastrointestinal) OR (Tumor, Gastrointestinal Stromal) OR (Tumors, Gastrointestinal Stromal) OR (Gastrointestinal Stromal Neoplasms) OR (Neoplasm, Gastrointestinal Stromal) OR (Neoplasms, Gastrointestinal Stromal) OR (Stromal Neoplasm, Gastrointestinal) OR (Stromal Neoplasms, Gastrointestinal) OR (Gastrointestinal Stromal Neoplasm) OR (Gastrointestinal Stromal Sarcoma))**

**Items found: 12742**

1. ****((Mesylate, Imatinib) OR (Imatinib Methanesulfonate) OR (Methanesulfonate, Imatinib) OR (STI571) OR (STI-571) OR (STI 571) OR (Gleevec) OR (Glivec) OR (ST 1571) OR (ST1571) OR (CGP 57148) OR (CGP57148B) OR (CGP-57148) OR (CGP57148) OR (Imatinib) OR (Alpha-(4-methyl-1-piperazinyl)-3'-((4-(3-pyridyl)-2-pyrimidinyl)amino)-p-tolu-p-toluidide))****

****Items found: 17524****

1. ****((Randomized controlled trials) OR (Clinical Trials, Randomized) OR (Trials, Randomized Clinical) OR (Controlled Clinical Trials, Randomized))****

****Items found: 794609****

1. ****1&2&3****

****Items found: 137****

****Web of Science****

1. **((gastrointestinal stromal tumor) OR (Stromal Tumor, Gastrointestinal) OR (Stromal Tumors, Gastrointestinal) OR (Tumor, Gastrointestinal Stromal) OR (Tumors, Gastrointestinal Stromal) OR (Gastrointestinal Stromal Neoplasms) OR (Neoplasm, Gastrointestinal Stromal) OR (Neoplasms, Gastrointestinal Stromal) OR (Stromal Neoplasm, Gastrointestinal) OR (Stromal Neoplasms, Gastrointestinal) OR (Gastrointestinal Stromal Neoplasm) OR (Gastrointestinal Stromal Sarcoma))**

**Items found: 20579**

1. ****((Mesylate, Imatinib) OR (Imatinib Methanesulfonate) OR (Methanesulfonate, Imatinib) OR (STI571) OR (STI-571) OR (STI 571) OR (Gleevec) OR (Glivec) OR (ST 1571) OR (ST1571) OR (CGP 57148) OR (CGP57148B) OR (CGP-57148) OR (CGP57148) OR (Imatinib) OR (Alpha-(4-methyl-1-piperazinyl)-3'-((4-(3-pyridyl)-2-pyrimidinyl)amino)-p-tolu-p-toluidide))****

****Items found: 37861****

1. ****((Randomized controlled trials) OR (Clinical Trials, Randomized) OR (Trials, Randomized Clinical) OR (Controlled Clinical Trials, Randomized))****

****Items found: 840427****

1. ****1&2&3****

****Items found: 266****

****Embase****

1. **((gastrointestinal stromal tumor) OR (Stromal Tumor, Gastrointestinal) OR (Stromal Tumors, Gastrointestinal) OR (Tumor, Gastrointestinal Stromal) OR (Tumors, Gastrointestinal Stromal) OR (Gastrointestinal Stromal Neoplasms) OR (Neoplasm, Gastrointestinal Stromal) OR (Neoplasms, Gastrointestinal Stromal) OR (Stromal Neoplasm, Gastrointestinal) OR (Stromal Neoplasms, Gastrointestinal) OR (Gastrointestinal Stromal Neoplasm) OR (Gastrointestinal Stromal Sarcoma))**

**Items found: 24518**

1. ****((Mesylate, Imatinib) OR (Imatinib Methanesulfonate) OR (Methanesulfonate, Imatinib) OR (STI571) OR (STI-571) OR (STI 571) OR (Gleevec) OR (Glivec) OR (ST 1571) OR (ST1571) OR (CGP 57148) OR (CGP57148B) OR (CGP-57148) OR (CGP57148) OR (Imatinib) OR (Alpha-(4-methyl-1-piperazinyl)-3'-((4-(3-pyridyl)-2-pyrimidinyl)amino)-p-tolu-p-toluidide))****

****Items found: 49915****

1. ****((Randomized controlled trials) OR (Clinical Trials, Randomized) OR (Trials, Randomized Clinical) OR (Controlled Clinical Trials, Randomized))****

****Items found: 339871****

1. ****1&2&3****

****Items found: 193****

****Cochrane Library****

1. **((gastrointestinal stromal tumor) OR (Stromal Tumor, Gastrointestinal) OR (Stromal Tumors, Gastrointestinal) OR (Tumor, Gastrointestinal Stromal) OR (Tumors, Gastrointestinal Stromal) OR (Gastrointestinal Stromal Neoplasms) OR (Neoplasm, Gastrointestinal Stromal) OR (Neoplasms, Gastrointestinal Stromal) OR (Stromal Neoplasm, Gastrointestinal) OR (Stromal Neoplasms, Gastrointestinal) OR (Gastrointestinal Stromal Neoplasm) OR (Gastrointestinal Stromal Sarcoma))**

**Items found: 547**

1. ****((Mesylate, Imatinib) OR (Imatinib Methanesulfonate) OR (Methanesulfonate, Imatinib) OR (STI571) OR (STI-571) OR (STI 571) OR (Gleevec) OR (Glivec) OR (ST 1571) OR (ST1571) OR (CGP 57148) OR (CGP57148B) OR (CGP-57148) OR (CGP57148) OR (Imatinib) OR (Alpha-(4-methyl-1-piperazinyl)-3'-((4-(3-pyridyl)-2-pyrimidinyl)amino)-p-tolu-p-toluidide))****

****Items found: 1590****

1. ****((Randomized controlled trials) OR (Clinical Trials, Randomized) OR (Trials, Randomized Clinical) OR (Controlled Clinical Trials, Randomized))****

****Items found: 8491****

1. ****1&2&3****

****Items found: 137****

****ClinicalTrials****

****Gastrointestinal Stromal Tumor + Imatinib failure****

****Items found: 32****

****Total: 765****

****After removing the duplicated records: 417****

****After selection based on titles and abstract: 24****

****Interim reports from same registered studies: 12****

****Second-line: 2****

****Not RCT: 2****

****Not TKI: 1****

****Eligible trails: 7****
